# Cell Proliferation of HaCaT Keratinocytes on Collagen Films Modified by Argon Plasma Treatment

**DOI:** 10.3390/molecules15042845

**Published:** 2010-04-20

**Authors:** Jorge López García, Ahmad Asadinezhad, Jiří Pacherník, Marián Lehocký, Ita Junkar, Petr Humpolíček, Petr Sáha, Pavel Valášek

**Affiliations:** 1 Polymer Centre, Faculty of Technology, Tomas Bata University in Zlín, T.G.M Sq. 275, 76272, Zlín, Czech Republic; E-Mails: vextropk@gmail.com (J.L.G.); asadinezhad@ft.utb.cz (A.A.); saha@utb.cz (P.S.); 2 Faculty of Sciences, Institute of Experimental Biology, Masaryk University Brno, Kotlářska 2, 61137, Brno, Czech Republic; E-Mail: jipa@sci.muni.cz (J.P.); 3 Tomas Bata University in Zlín, T.G.M Sq. 5555, 76001, Zlín, Czech Republic; E-Mails: humpolicek@uni.utb.cz (P.H.); valasek@ft.utb.cz (P.V.); 4 Plasma Laboratory, Department of Surface Engineering, Jožef Stefan Institute, Jamova cesta 39, SI-1000, Ljubljana, Slovenia; E-Mail: ita.junkar@ijs.si (I.J.)

**Keywords:** plasma treatment, atelocollagen, surface modification, HaCaT, cell proliferation

## Abstract

Argon plasma treatment was used to modify the surface of atelocollagen films using a plasmochemical reactor. To evaluate the effects of the treatment, the untreated and treated samples were characterized by Attenuated Total Reflectance Fourier Transform Infrared Spectroscopy (ATR-FTIR), Scanning Electron Microscopy (SEM) imaging, and X-ray Photoelectron Spectroscopy (XPS) techniques. Cell growth was carried out by culturing human immortalized keratinocyte (HaCaT) cells and proliferation was measured via MTT assay. It was observed that argon plasma treatment significantly enhanced the extent of cell proliferation, which was ascribed to the favourable role of plasma treatment in inducing surface oxygen-containing entities together with increasing surface roughness. This can be considered as a potentially promising approach for tissue regeneration purposes.

## 1. Introduction

Collagen is a fibrillar protein that exists in nearly all mammalian tissues. It constitutes *ca.* 25% of whole-body protein content. Its abundance is especially concentrated in connective tissues such as tendons, ligaments, cartilage as well as skin. Moreover, it is connected with important biological functions such as tissue formation and cell attachment [1]. On this account, collagen is extensively used in the design of materials with potential applications in the biomedical field. 

Skin consists of different types of cells such as keratinocytes, melanocytes, and fibroblasts [2]. It is well established through wound healing, transplantation, and cell culture studies that human immortalized keratinocyte (HaCaT) cells play a crucial role in epidermal tissue regeneration, since they are spontaneously transformed to human keratinocytes which have the traits of basal epidermal keratinocytes and can be delivered in deep burns. Hence, this cell line can be exploited as an *In vitro* model for highly proliferative epidermis [3,4,5,6]. 

Argon is a member of the noble gases class which is a group of chemical elements of identical properties. They are colourless, odourless, and possess very low reactivity because of a full valence shell [7]. For this reason, plasma treatment by noble gases is of importance since these gases do not chemically react with the treated sample. Nevertheless, it conveys reactivity onto the treated surface via plasma species, electrons, ions, and UV-radiation. In fact, inert gas plasma treatment is used in periods typically from 1s to several minutes, and this exposure is enough to abstract hydrogen and to produce free radicals at or near the surface which then interact to form cross-links and unsaturated groups; notwithstanding, these chemical and physical changes are restricted to the top several hundred angstroms without affecting bulk properties [8,9].

Depending upon the noble gas and time of the treatment, this type of plasma treatment can then be performed for cleaning, sputtering, etching, implantation, and deposition on the substrates; for example, helium, neon and argon are often used for cleaning and sputtering, while argon, krypton, and xenon have found applications in implantation and deposition. Nonetheless, argon is the most common noble gas used in plasma treatment due to its relatively low cost, availability, and high yield [10]. 

Noble gases-based plasma treatment has widely been used in a diversity of applications, e.g., surface modification of polymers [11,12,13,14,15,16,17], glass [18], carbon fibres [19], superconductors [20], metal and alloys [21,22], and textiles [23,24,25,26]. As for medical uses, there are several studies conducted on cleaning surfaces [27,28,29] and cell attachment [30,31,32,33,34,35]. In this contribution, focus is directed onto the surface modification via argon plasma treatment and examination of keratinocyte human cell growth on atelocollagen surfaces which has not yet been done. Furthermore, a systematic study of argon plasma treatment effects on HaCaT keratinocyte cell response of atelocollagen films with a view to designing a novel material potentially suitable for tissue engineering applications is undertaken. This is accomplished via surface probe techniques together with pertinent biological assays.

## 2. Results and Discussion

### 2.1. Surface Spectroscopic Analysis

X-ray photoelectron Spectroscopy (XPS) spectra have been recorded in order to gain an insight into the chemical modifications of the treated surface. From the analysis of these spectra, carbon (C1s), oxygen (O1s), nitrogen (N1s), and sulphur (S2p) elements were detected on both untreated and argon plasma treated samples surfaces. The respective elemental compositions along with the corresponding atomic ratios are shown in Table 1. The data shows a considerable increase in the oxygen content subsequent to the argon plasma treatment which is also reflected as a raise in O/C ratio. This is ascribed to the occurrence of surface oxidizations which are, stimulated by the argon plasma treatment followed by exposure to the air [36,37,38,39]. As suggested by the data, nitrogen content slightly decreases which can be connected with the etching phenomenon, while that of sulphur remains unchanged. The presence of sulphur seems to stem from sulphur-containing amino acids [40,41].

**Table 1:** Elemental compositions of the untreated and treated films by XPS measurement.

Sample	C1s%	N1s%	O1s%	S2p%	N1s/C1s	O1s/C1s
**Untreated samples**	66.0	14.1	19.3	0.4	0.21	0.29
**Argon plasma treatment**	58.1	11.3	25.0	0.4	0.19	0.43

**Figure 1:**
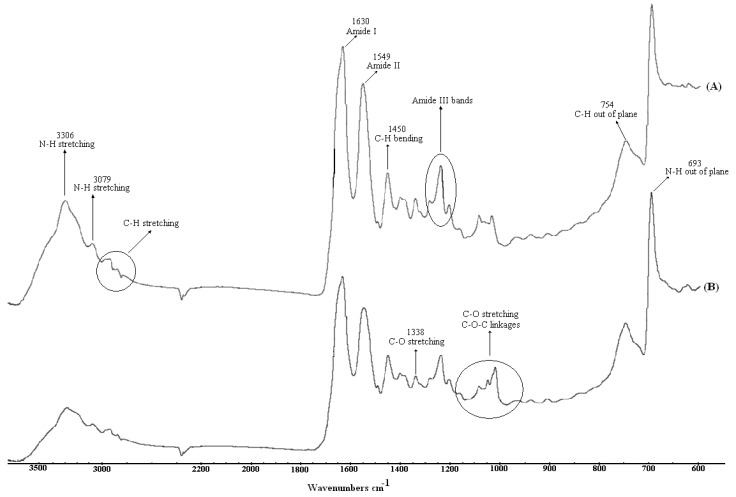
ATR-FTIR spectra of: (A) untreated and (B) argon plasma-treated films.

Attenuated Total Reflectance Fourier Transform Infrared (ATR-FTIR) spectra from untreated and argon plasma treated samples are shown in Figure 1. In the untreated sample spectrum, amide I and II characteristic bands at 3,306, 3,079, 1,630, and 1,549 cm^−1^ can be identified, where the ones at 3,306 and 3,079 cm^−1^ correspond to peptide bond N-H stretching. The C=O stretching interaction with the amide I N-H vibration gives rise to an absorption at 1,630 cm^−1^, while its interaction with C-N leads to the band at 1,549 cm^−1^. Characteristic signals due to amide III appear at 1,280, 1,239, and 1,204 cm^−1^ which originate from N-H bending and C-N stretching interactions. In addition, in the spectrum of the untreated sample, one can recognize the other typical amide vibrations assigned to C-N stretching and N-H wagging which appear at 1,400 and 693 cm^−1^, respectively [42,43]. The absorption peaks within the 3,000-2,800 cm ^−1 ^spectral range are attributed to aliphatic C-H stretching, likewise, the bands at 1,491, 1,450, and 851 cm^−1 ^are associated with C-H vibrations. As for the argon plasma treated sample, the characteristic amide bands are also visible which implies that atelocollagen is retained upon plasma treatment. However, the intensity of the bands decreases after the treatment which can be a consequence of plasma-induced reactions [44,45]. Furthermore, the bands within 1,160–1,000 cm^−1^, assigned to C-O stretching mode, undergo evident alterations after argon plasma treatment. Particularly, a peak at around 1,100 cm^−1 ^ due to C-O-C linkage gains strength, which means that argon plasma treatment affects the chemical composition of the surface, as earlier discussed in more detail in XPS section. It should also be noted that unsaturated double bonds such as alkenes (-C=C-) and imino (-C=N-) can possibly arise after the argon plasma treatment [46,47], but cannot be viewed due to overlapping peaks, prospective cross-linking or the insufficient surface-sensitivity of ATR-FTIR. 

**Figure 2:**
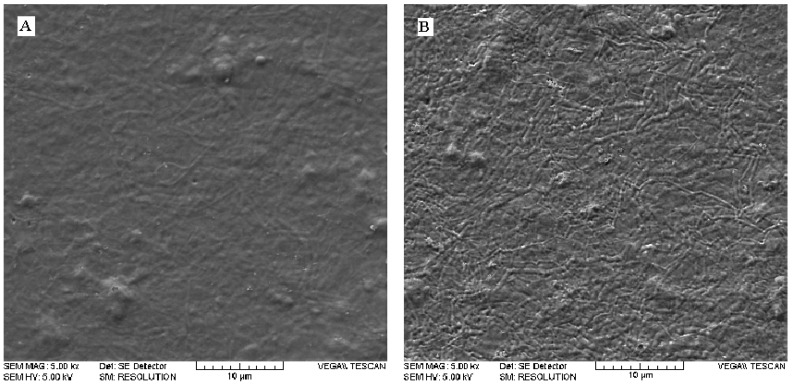
SEM surface topography of (A) untreated and (B) argon plasma-treated samples.

### 2.2. Effects of argon plasma upon surface topography

Visible changes in surface topography can be observed after argon plasma treatment, as evident from Scanning Electron Microscopy (SEM) images (Figure 2). The untreated film has a relatively smooth morphology, while that of argon plasma treated sample is of higher roughness and shows etched features. This is in agreement with the results mentioned in previous sections, where surface etching due to the argon plasma treatment was observed. An enhanced roughness is considered as a beneficial factor in adhesion processes, since it is a consequence of surface etching and functionalization [48].

### 2.3. Effects of argon plasma treatment on cellular behaviour of HaCaT on collagen films

**Figure 3:**
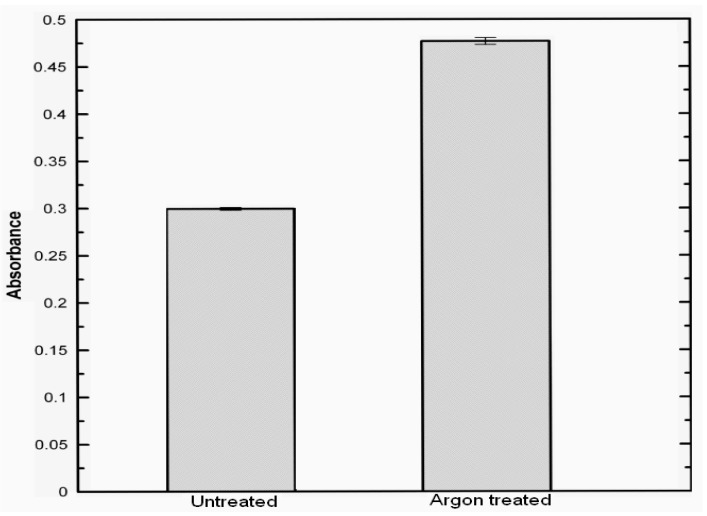
Comparison of HaCaT cells growth upon untreated and argon plasma-treated films, measured by MTT cell proliferation assay at 570 nm.

**Figure 4:**
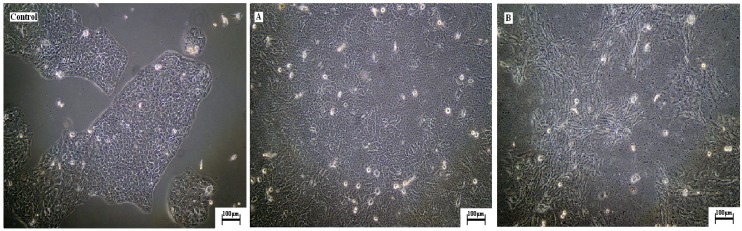
Light micrographs of Human skin HaCaT keratinocytes in culture upon the collagen films compared with control, (A) untreated, (B) argon plasma-treated.

HaCaT cells behaviour on untreated and argon plasma treated samples evaluated using MTT assay is given in Figure 3. It is found out that cell attachment significantly increases, as reflected by the absorbance value which is approximately 1.6 times higher for the argon plasma treated samples than the untreated films. This indicates that argon plasma treated films show higher cell-substrate compatibility and thus, are more appropriate for tissue regeneration applications. This is also supported qualitatively by the light microscope images shown in Figure 4, where the extent of cell adhesion on untreated and argon plasma treated samples can be compared to a control specimen. A higher amount of cell aggregates in form of ripple-like areas adhered on the surface is identified for argon plasma treated sample. Although the mechanisms of the HaCaT adhesion and proliferation upon the different substrates are still unclear, it is well known that roughness and porosity of films influence cell adhesion and cell proliferation. Besides, surface polar entities content is a crucial factor because HaCaT cells are mainly attached by carbonyl and carboxyl groups, thus their cell growth tends to be favoured in hydrophilic surfaces [49,50,51,52,53]. This attachment is also supported by hydrogen bonding and van der Waals forces, which reinforce the linking between cells and films [54]. An increase in roughness and surface polar functionalities after exposing atelocollagen films to argon plasma promote cell adhesion and proliferation. The results suggest that argon plasma treated atelocollagen films are potentially suitable materials for tissue regeneration.

## 3. Experimental

### 3.1. Materials

Atelocollagen from bovine Achilles tendon (emulsion which contents 1.43% of atelocollagen, pH 3.5) was supplied by Vipo A.S, Slovakia. Tissue culture dishes of diameter 40 were provided by (TPP, Switzerland). Acetic acid 99% was obtained from Penta, Czech Republic. Vybrant^®^ MTT cell proliferation Assay kit (V-13154) was purchased from Invitrogen Corporation, USA. Human immortalized non-tumorigenic keratinocyte cell line HaCaT [55] (Ethnicity, Caucasian; Age, 62 years; gender, Male and tissue, skin) was supplied by CLS Cell Lines Service, Germany. DMEM (high glucose) supplemented with 2 mM L-glutamine solution and 10% fetal calf serum were provided by Biotech Inc, USA, which was used as the culture medium for HaCaT cell line.

### 3.2. Preparations of collagen films

The atelocollagen was solubilized in 0.1M acetic acid to prepare a 0.1%w/w solution using an IKA RCT stirring machine (IKA^®^ works, Inc, Germany) for 4 hours at 1,000 rpm. Then, 2 mL of this solution was poured into each of the tissue culture dishes. Thereafter, the solvent was evaporated at ambient conditions for three days.

### 3.3. Plasma treatment

The plasma treatment of the collagen thin films was carried out by using plasmochemical reactor (Femto, Diener electronic, Germany) with a chamber of 100 mm diameter and 270 mm length, operated at frequency of 40 kHz, pressure of 40 Pa, and power input of 50 W. Argon grade 4.5 was used as carrier gases provided by Linde, A.G., Germany. The feed rate in all experiments was 5 cm^3^/min. The duration of the plasma treatment was 5 minutes for each sample. 

### 3.4. Spectroscopic techniques

Surface chemical composition of both untreated and treated collagen films were evaluated by XPS which was performed in a XPS microprobe instrument PHI Versaprobe (Physical Electronics, USA). The base pressure in the XPS analysis chamber was about 6×10^−8^ Pa. The samples were excited with X-rays over a 400 μm spot area with a monochromatic Al *K*_α1,2_ radiation at 1,486.6 eV. The photoelectrons were detected with a hemispherical analyzer positioned at an angle of 45° with respect to the normal of the sample surface. The energy resolution was about 0.5 eV and survey-scan spectra were made at 187.85 eV. Individual high-resolution C1s and O1s spectra were taken at 23.5 and 0.1 eV energy step for 30 minutes and the concentration of different chemical states of carbon in the C1s peak was determined by fitting the curves with symmetrical Gauss–Lorentz functions. The spectra were fitted using MultiPak v7.3.1 software from Physical Electronics, USA; which was supplied with the spectrometer. Attenuated total reflection Fourier transform infrared spectroscopy (ATR-FTIR) was carried out on a FTIR spectrometer Avatar 320 (Nicolet, USA) equipped with ATR accessory. Each spectrum was obtained by recording 32 scans at a 2 cm^−1 ^resolution. The spectral range was within 4,000–650 cm^−1^.

### 3.5. Microscopic techniques

The surface morphology of collagen untreated and treated films were analyzed by using SEM on a Vega LMV microscope (Tescan s.r.o, Czech Republic) operated at 5 kV. The specimens were 30^°^ tilted to attain higher resolution and observation. All of samples were coated with a thin layer of Gold/Palladium alloy. The images were taken at magnifications of 5,000 ×. 

### 3.6. Cell culture

HaCaT keratinocyte cells were seeded onto the treated and untreated samples in the culture dishes and incubated at 37 °C for 4 days. The cell culture was performed in 32 tissue culture dishes, 16 for untreated films and 16 for argon plasma treated films.

### 3.7. Cell proliferation

The HaCaT cell proliferation on treated and untreated films was determined after 4 days of culturing by MTT assay [reduction of 3-(4,5-dimethylthiazol-2-yl)-2,5-diphenyltetrazolium bromide, which is yellow, to a purple formazan product]. A volume of 10 μL of 12 mM MTT was taken for cell incubation performed at 37 °C for 4 hours in the darkness. The media were then decanted and washed with phosphate-buffered saline solution (PBS). The produced formazan salts were dissolved with dimethylsulphoxide (DMSO, Sigma-Aldrich, USA), and the absorbance was measured at 570 nm to estimate the formazan concentration [56]. The statistical analysis of the recorded data was managed using Student’s *t*-test, where a confidence level of 95% (*p < *0.05) was considered statistically significant and 99% (*p < *0.01) was considered very significant.

## 4. Conclusions

The effects of argon plasma treatment upon atelocollagen surface films and HaCaT cell response have been studied by means of surface probe techniques together with the biological assay. It is assumed that the primary effect of argon plasma treatment is to provide and deposit energy via plasma species such as electrons, ions and UV-radiation to the substrate surface leading to cross-linking, bond breaking and different kind of intramolecular rearrangements, which in contact with air or other reactive species can drastically alter the surface properties. The spectroscopic techniques show a decrease of the nitrogen content along with the attenuation of the N-H and C-H bands, which can be attributed to the etching phenomenon. It is observed that argon plasma treatment significantly enhances the extent of cell proliferation ascribed to the favourable role of plasma treatment in inducing surface oxygen-containing entities and increasing surface roughness. The keratinocyte HaCaT cell proliferation notably increases confirming the strong dependence of cell adhesion and proliferation on the surface properties and biocompatibility.
